# Primary hepatic diffuse large B cell lymphoma: A case report: Primary hepatic diffuse large B cell lymphoma

**Published:** 2011-03-01

**Authors:** Yuan-Ji Ma, En-Qiang Chen, Xue-Bing Chen, Juan Wang, Hong Tang

**Affiliations:** 1 Center of Infectious Diseases, West China Hospital of Sichuan University, Sichuan Province, China

**Keywords:** Primary hepatic lymphoma, Diffuse large B cell lymphoma, Chemotherapy, Neoplasm

## Abstract

In this report we describe a rare case of primary hepatic diffuse large B cell lymphoma in a 67-year-old man who presented with abdominal pain, deteriorated liver function, elevated lactate dehydrogenase. He was found to have diffuse nodular intrahepatic space-occupying lesion with normal α-fetoprotein and carcino-embryogenic antigen. The final diagnosis was made by percutaneous biopsy of the liver as the clinical manifestation not consistent with common liver diseases. The patient was treated with R-CHOP (rituximab, cyclophosphamide, doxorubicin, vincristine and prednisone) without surgical resection with a favorable response. However, serious complication was occurred after 4 cycles of chemotherapy, and the patient finally died of concurrent acute respiratory distress syndrome.

## Introduction

Primary hepatic lymphoma (PHL) is an unusual form of non-Hodgkin's lymphoma that usually presents with constitutional symptoms, hepatomegaly and signs of cholestatic jaundice without lymph node and extrahepatic (.i.e., the spleen, bone marrow and other lymphoid tissue) lymphoma proliferation at early stage of the disease [1].The prevalence of PHL was 0.4% among extranodal non-Hodgkin's lymphoma, and 0.016% among all non-Hodgkin's lymphoma [2]. Because of its rarity, non-specific clinical symptoms, laboratory and imaging performance, PHL was often misdiagnosed as hepatitis, primary liver cancer or metastatic tumor. In this case report, we present a patient with pathologically confirmed primary hepatic diffuse large B cell lymphoma.

## Case presentation

**Figure 1:**
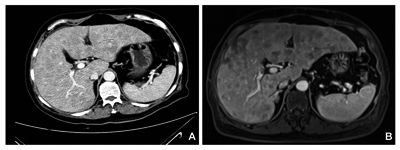
Abdominal computed tomography (A) and T1-weighted magnetic resonance imaging (B), revealing multiple occupying nodules in liver. Neither splenomegaly nor abdominal lymphadenopathy was observed.

A 67-year-old Chinese man was hospitalized for fatigue, anorexia, yellowish discoloration of skin and sclera, and right upper quadrant abdominal pain. The patient had no complaints of fever, significant weight loss. He did not have history of smoking, alcohol consumption and drfugs abuse. He had a history of cholecystectomy at the age of 45. Physical examination revealed no abnormal findings except for tenderness on percussion on liver anfd edema in both lower extremities. There was no palpable superficial lymphadenopathy. He had no hepatosplenomegaly. Laboratory findings showed deteriorated liver function, elevated blood lipids and lactate dehydrogenase (LDH) (Table 1). Serologic tests for hepatitis A, B and C were negative; serum α-fetoprotein (AFP) and carcinoembryonic antigen (CEA) levels were normal. Immunoglobulin levels were normal without any monoclonal peak in the plasma or urine electrophoresis. Abdominal ultrasound and computed tomography (CT) revealed diffuse occupying nodules in liver (Figure 1A). Subsequent magnetic resonance imaging (MRI) revealed diffuse lesions that were hypointense on T1 and hyperintense on T2-weighted images (Figure 1B).

**Figure 2:**
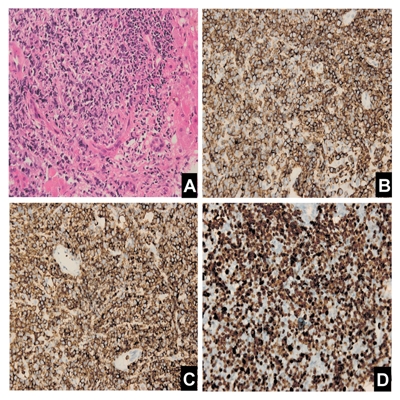
Histological and immunohistochemical photographs of liver tumor. (A): Hematoxylin and eosinstaining, 400×; (B): anti-CD20 staining, 400×; (C): anti-LCA staining, 400×; (D): anti-ki67 staining, 400×

There was no evidence of biliary or pancreatic disease, splenomegaly or abdominal lymphadenopathy. Bone marrow biopsy demonstrated normal proliferation and maturation of all cell lines. There was no malignant infiltration; flowcytometry examination also showed no obvious abnormality of bone marrow. A core liver biopsy was performed, showing heavy infiltration composed mainly of medium-sized round cells (Figure 2A). The cells were positive for CD20 and LCA (Figures 2B and Figures 2C), and proliferation index was very high with ki67 positive in 90% of cells (Figure 2D), establishing a diagnosis of diffuse large B-cell lymphoma, according to the WHO classification.

After discussing treatment options and the risks of chemotherapy with the patient and her family, the patient was then received attenuated chemotherapy (R-CHOP: rituximab 500 mg, cyclophosphamide 400 mg, doxorubicin 40 mg, vincristine 2 mg, and prednisone 60 mg). The courses of chemotherapy were given every 2-3 weeks. After receiving two more cycles of the chemotherapy (rituximab 500 mg, cyclophosphamide 800 mg, doxorubicin 60 mg, vincristine 2 mg and prednisone 60 mg), his liver function was improved-the level of LDH was decreased (Table 1), and the intrahepatic diffuse tuberculous lesions were decreased both in number and size. However, after the 4th cycle of the chemotherapy, the patient developed neutropenia, diarrhea, followed by intestinal bleeding, signs of peritonitis, and pneumonia. Unfortunately, his condition got worse rapidly, and the patient finally died of concurrent acute respiratory distress syndrome (ARDS).

**Table 1:** Laboratory test results

**Item**	**Weeks after the first admission**					**Reference Values**	**Unit**
	**0**	**2**	**3 a**	**6 b**	**9 c**		
**Total bilirubin**	117.7	286.8	356.8	115.0	20.4	2.0-28.0	µmol/L
**Direct bilirubin**	97.1	232.4	278.5	93.0	15.0	< 8.8	µmol/L
**Alanine transarninase**	79	93	109	57	14	< 55	U/L
**Aspartate transarninase**	139	243	291	54	32	< 46	U/L
**Alkaline phosphatase**	467	1025	1492	435	127	47-138	U/L
**ɣ-glutamyl transferase**	637	595	827	342	117	6-46	U/L
**Albumin**	32.5	26.1	23.6	30.9	34.5	35-55	g/L
**Globulin**	36.4	38.6	39.7	30.2	20.3	19-34	g/L
**Triglyeride**	1.89	2.84	3.16	3.19	1.88	0.29-1.83	mmol/L
**Cholesterol**	6.13	11.06	14.22	10.10	3.07	2.8-5.7	mmol/L
**Lactate dehydrogenase**	434	513	448	197	211	110-220	U/L

^a^ Laboratory results before the first chemotherapy

^b^ Laboratory results before the second chemotherapy

^c^ Laboratory results before the third chemotherapy

## Discussion

Non-Hodgkin's lymphoma is a common malignant disease. Liver involvement occurs in 10% of patients and is a sign of advanced disease. PHL refers to an extra-nodal lymphoma of the liver without involvement of any other organ (e.g., lymph node, spleen, etc) [3][4]. The vast majority (67%) of PHL patients are middle-aged men who usually present with abdominal pain, nausea and constitutional symptoms [4]. PHL is notably rare, representing <1% of all extra-nodal lymphoma [4]. One Chinese study reported that in 446 cases of non-Hodgkin's lymphoma, 45 developed liver involvement, of which only one had PHL [5]. Limited experience showed that PHL had non-specific clinical manifestations. Hepatomegaly is found in most patients, constitutional symptoms (e.g., fever, night sweats and weight loss) appear in 37%, fever in 86%, weight loss in 57% and jaundice in 4% [3][6]. In liver, PHL may present as a solitary mass (42%) or as multiple lesions (50%). Patients with PHL have abnormal liver function tests (cholestasis and cytolysis) [7][8], mostly elevated LDH and alkaline phosphatase (ALP) [6][9]. Additionally, hypercalcemia is found in 40% of the patients. The cause of PHL is not entirely clear, but may be related to viral hepatitis [10][11]. Hepatitis C virus (HCV) infection is found in 20%-60% of patients with PHL. The frequent association with HCV suggests that this virus may play a role in the pathogenesis of PHL [7][8]. PHL is also seen in immunocompromised patients, but the relationship between PHL occurrence and immune deficiency has not yet been reported. Our patient had neither hepatitis C infection nor signs of immunodeficiency. Therefore, we speculated that PHL also could occur in patients without any prior liver disease. Diagnosis of PHL requires a liver biopsy compatible with lymphoma and absence of lymphoproliferative disease outside the liver. Through analyzing 90 patients with hepatic lymphoma, Lei Ki [4] proposed the following criteria for the diagnosis of primary hepatic lymphoma: 1) the symptoms are mainly caused by liver involvement at presentation; 2) no clear evidence of superficial lymph node enlargement and distant lymph node metastasis; 3) no abnormalities in peripheral blood cells in blood smears, including spleen, lymph node and bone marrow. Some other studies also reported that the dynamic change of serum LDH could be used as a diagnostic marker [12]. However, the value of LDH for the diagnosis of PHL is limited because of its poor specificity. Therefore, a definite diagnosis of PHL is difficult to establish solely based on clinical signs and symptoms. The imaging presentation of PHL is variable and can be a solitary intrahepatic lesion, multiple nodules and diffuse infiltration of the liver. In the imaging reports of 12 patients with PHL provided by Elsayes KM, et al [13], there were three cases of solitary nodules, one with diffuse damage, and eight with multiple nodular liver lesions. Multiple nodular lesions in liver accounted for the majority of these cases, while diffuse infiltration was rare. Imaging of our patient showed diffuse nodular lesions, and the presentation varied in ultrasound, CT or MRI. Therefore, there is no pathognomonic imaging pattern to confirm the diagnosis.

Hepatoma and metastasis from gastro-intestinal (mostly colon) carcinoma present very similarly and are much more common. Normal levels of the tumor markers, AFP and CEA are found in almost 100% of patients with PHL facilitating the differential diagnosis [3][4][6]. The examination of the colon to exclude primary colon carcinoma may be indicated. Thus, a definite diagnosis of PHL should include histological and marker studies of the biopsy sample. Our patient presented with clinical and laboratory features which were suggestive for PHL; liver biopsy stained with specific immuno-histochemical stains and flowcytometric studies also confirmed the diagnosis of PHL. There is no consensus on the optimal treatment for PHL. Surgical treatment, radiotherapy and chemotherapy were all reported as treatment modalities alone or in combination. The prognosis of PHL is considered very poor with a median survival as low as six months for patients treated with chemotherapy alone. With the availability of rituximab (a monoclonal chimeric antibody directed against CD20 B cell antigen), chemotherapy protocols for the treatment of PHL have dramatically changed in the last decade. R-CHOP protocol increased the complete-response rate and prolonged the survival significantly. Unfortunately, our patient was died of complications of chemotherapy. Our report indicated that complications caused by chemotherapy still would be a major problem.

In conclusion, PHL is a rare disease, lacking specific imaging and clinical manifestations and biochemical indicators. Its diagnosis is difficult, needing to exclude organs or tissues lymphoma outside of the liver. When multiple space-occupying lesions are found in liver but there is no any other organ or tissue invasion, PHL should be suspected and liver biopsy should be done. If the diagnosis is made, chemotherapy should be started immediately, and adverse reactions and complications should be closely monitored.
